# Cancer-associated fibroblast-derived extracellular vesicles: regulators and therapeutic targets in the tumor microenvironment

**DOI:** 10.20517/cdr.2024.152

**Published:** 2025-01-07

**Authors:** Jindong Xie, Xinmei Lin, Xinpei Deng, Hailin Tang, Yutian Zou, Wenkuan Chen, Xiaoming Xie

**Affiliations:** ^1^State Key Laboratory of Oncology in South China, Guangdong Provincial Clinical Research Center for Cancer, Sun Yat-sen University Cancer Center, Guangzhou 510060, Guangdong, China; ^2^School of Medicine, Sun Yat-sen University, Guangzhou 510080, Guangdong, China.; ^#^Authors contributed equally.

**Keywords:** Cancer-associated fibroblasts, extracellular vesicle, tumor microenvironment, therapeutic targets

## Abstract

Cancer-associated fibroblasts (CAFs) constitute a critical component of the tumor microenvironment (TME). CAFs can be reprogrammed by cancer cells, leading to the production of extracellular vesicles (EVs). These EVs serve as carriers for bioactive substances, including proteins, nucleic acids, and metabolic products, thereby facilitating tumor progression. CAF-derived EVs exert substantial influence on tumor cell proliferation, invasion, and metastasis, the immunological environment, and the processes of lymphangiogenesis and angiogenesis. Despite their potential as non-invasive biomarkers and therapeutic delivery vehicles, the clinical application of CAF-derived EVs is currently limited by challenges in purification and precise targeting. This review delineates the diverse roles of CAF-derived EVs in tumor growth, metastasis, and immune evasion within the TME.

## INTRODUCTION

The tumor microenvironment (TME) refers to the internal milieu within which tumor cells proliferate and subsist, which consists of various cellular components and non-cellular components. Cellular components consist of immune cells and stromal cells. Among the immune cells are T and B lymphocytes, tumor-associated macrophages (TAMs), dendritic cells, natural killer cells, neutrophils, and myeloid-derived suppressor cells (MDSCs), and so forth. Stromal cells comprise tumor-associated fibroblasts [cancer-associated fibroblasts (CAFs)], pericytes, endotheliocytes, mesenchymal stromal cells, and the like; non-cellular components encompass extracellular matrix (ECM) and other secreted molecules such as growth factors, cytokines, chemokines, and extracellular vesicles (EVs), as well as the blood and lymphatic vascular network^[1]^. The composition of the TME is quite complex and is a dynamic and variable process. It was once considered bystanders of tumorigenesis but is now known to play critical roles in the pathogenesis of cancer. However, mechanistic studies, including those involving preclinical tumor models, have shown that cancer development and progression occur alongside alterations in the surrounding stroma. Cancer cells and all other cells in the TME can functionally reprogram their microenvironment by secreting various cytokines, chemokines, and other factors, enabling them to play a determinative role in tumor survival and progression. The cellular composition and functional state of the TME can differ extensively depending on the organ in which the tumor arises, the intrinsic features of cancer cells, the tumor stage, and patient characteristics^[1-3]^. In recent years, researchers have been exploring a new cancer treatment modality: targeting the tumor stroma.

CAFs are fibroblasts found within the TME near cancer cells and consist of multiple subtypes with distinct functions, demonstrating significant plasticity. Research suggests that CAFs are derived from different origins, such as pre-existing quiescent stellate cells and normal fibroblasts (NFs), bone marrow-derived fibroblasts and mesenchymal stem cells (MSCs), endothelial cells, epithelial cells, along with pericytes, smooth muscle cells, and adipocytes^[4]^. Based on the expression of certain markers, classifications of CAFs broadly converge on three main subtypes: myofibroblastic CAFs (myCAFs), inflammatory CAFs (iCAFs), and antigen-presenting CAFs (apCAFs). These subtypes undergo alterations during tumor progression and are regulated at the spatial level. For instance, in pancreatic cancer, three distinct CAF subtypes coexist and possess different functional characteristics and transcriptomic plasticity. The functions of myCAFs and iCAFs rely on the secretion of ECM and immunomodulatory factors, respectively, while apCAFs interact directly with T cells to promote T cell exhaustion^[2,5]^. CAFs are activated by inflammatory mediators within the TME, encompassing soluble factors generated by the tumor, such as transforming growth factor beta (TGF-β), interleukin-1 (IL-1), interleukin-6 (IL-6), and tumor necrosis factor-alpha (TNF-α)^[6,7]^. Furthermore, cancer cells can transform dormant NFs into CAFs through direct cell-to-cell communication. For example, cancerous pre-mammary ductal cells can also activate CAFs in situ through direct epithelial-stromal interactions mediated by Jagged1/Notch2 ligand-receptor binding^[8]^. Meanwhile, CAFs synthesize and remodel the ECM, modify the mechanical properties of the ECM, and alter the behaviors of cancer cells and immune cells. Additionally, CAFs have an impact on angiogenesis, possess robust immunomodulatory capabilities, and contribute to the immune evasion of cancer cells. CAFs interact extensively with cancer cells and can influence other components of the TME, such as the ECM and the immune infiltrates. Histopathological analysis has shown that the abundance of CAFs is related to prognosis among different human cancers^[6,7,9,10]^. CAFs also modulate the efficacy of therapies and constitute a therapy target in their own right.

EVs are particles released from cells that are enclosed by a lipid bilayer and do not contain functional nuclei. EVs can be derived from a variety of eukaryotic cells, such as tumor cells, immune cells, and stem cells, as well as prokaryotic cells, including Gram-positive and Gram-negative bacteria. Moreover, EVs can be isolated from multiple body fluids and solid tissues, including blood, urine, cerebrospinal fluid, saliva, synovial fluid, and brain or tumor tissues^[11,12]^. EVs have a broad range of biological functions and participate in multiple physiological and pathological processes. Their ability to mediate intercellular communication by transferring a wide spectrum of molecules between cells gives them an important role in complex biological processes such as tumorigenesis, inflammation, immune response modulation, tissue repair, and apoptosis^[13-15]^. In recent years, there has been growing evidence that CAFs are an important source of EVs in the TME, and CAF-derived EVs have been recognized as crucial mediators in regulating the extracellular communication between CAFs and cancer cells, and can affect tumor progression in multiple ways^[16,17]^.

## OVERVIEW OF EVs

According to the Minimal information for studies of extracellular vesicles 2023 (MISEV2023) guidelines, EVs can be divided into different subtypes based on their size, density, molecular composition, or cell origin, among other characteristics. For example, EVs can be classified into small EVs and large EVs based on the diameter of the separated particles. Small EVs are often described as < 200 nm in diameter. Additionally, EVs can be classified into exosomes and ectosomes based on their biogenesis pathways. Exosomes are EVs from internal compartments of the cell that are released via the multivesicular bodies (MVBs), while ectosomes are EVs from the cell surface. Additionally, ectosomes are enriched for CD9 and CD81, while exosomes are enriched in CD63, CD9, CD81, Alix, and syntenin^[11,18,19]^. Owing to the difficulty in isolating pure exosomes from cells in most instances and the ambiguity regarding the source of the isolated EVs, we term the exosomes mentioned in research articles “small Evs” or “Evs” rather than “exosomes”.

### Synthesis and release of EVs

**Figure 1:**
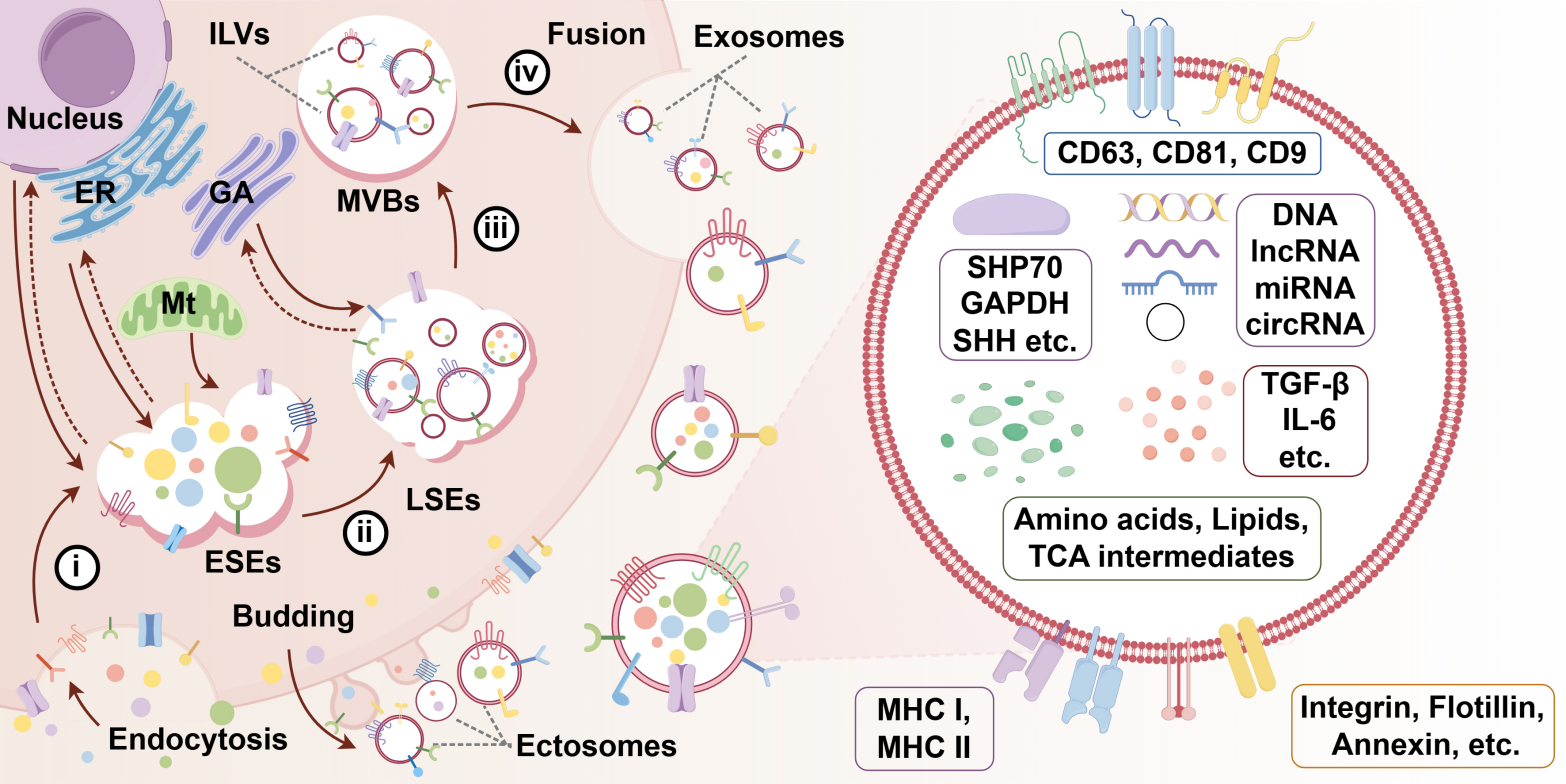
Biogenesis of extracellular vesicles and the structure and contents of exosomes. The formation of exosomes included four steps: (i) ESEs formation: Through the mechanisms of endocytosis and invagination of the plasma membrane, ESEs are formed, a process energized by Mt; (ii) LSE formation: After cytoplasmic sorting, ESEs mature and eventually form LSEs. During the formation of ESEs and LSEs, they are capable of exchanging materials with the cell nucleus, ER, and GA; (iii) MVB formation: LSEs’ membrane buds inwardly to form multiple ILVs (future exosomes), which eventually transform into MVBs; (iv) Release: MVBs fuse with the plasma membrane, and ILVs released to extracellular space are called exosomes. Ectosomes are vesicles that pinch off the surface of the plasma membrane via outward budding. Exosomes have a diameter of 30-150 nm and a bilayer membrane structure, while ectosomes range from 50 to 1 mm in diameter. EVs are highly heterogeneous bilayer membrane structures carrying diverse cargos, such as proteins, nucleic acids, and lipids, and their content can vary significantly across different cells and conditions. ESEs: Early sorting endosomes; Mt: mitochondria; LSEs: late sorting endosomes; MVBs: multivesicular bodies; ILVs: intraluminal vesicles; ER: endoplasmic reticulum; GA: Golgi apparatus; SHH: Sonic Hedgehog; Evs: extracellular vesicles; TGF-β: transforming growth factor beta; IL-6: interleukin-6; TCA: tricarboxylic acid.

Exosomes originate from the endosomal pathway by the formation of the early sorting endosomes (ESEs), late sorting endosomes (LSEs), and ultimately MVBs, which contain intraluminal vesicles (ILVs). Finally, MVBs fuse with the plasma membrane, and exosomes are released. Through endocytosis and plasma membrane invagination, fluid and extracellular constituents such as proteins, lipids, metabolites, small molecules, and ions can enter cells, along with cell surface proteins. However, ectosomes are vesicles that pinch off the surface of the plasma membrane via outward budding, and include microvesicles, microparticles, and large EVs in the size range of 50 to 1 mm in diameter. During EV synthesis, donor cells’ cytoplasm is loaded into EVs and secreted into the extracellular environment^[20-23]^ [Figure 1]. After successfully released into the extracellular environment, EVs can be taken up by interacting with receptors and ligands, thereby mediating material transport and information transmission between the TME and tumor cells^[24-26]^.

### Contents and functions of EVs

EVs are highly heterogeneous, which is reflected in differences in their contents. EVs can carry various cargoes, including proteins, nucleic acids, and lipids, and this content can vary widely between cells and conditions. In addition to carrying bioactive contents, EVs are also involved in tumor cell proliferation, metastasis, drug resistance, and immune response^[27-29]^ [Figure 1].

Proteins are important components of EVs contents. There are multiple protein families in EVs, such as receptors, transcription factors, enzymes, and ECM proteins^[30]^. EVs from different sources have different characteristics^[31]^. Proteins in EVs from CAFs can participate in tumorigenesis by activating signaling pathways in recipient cells. For example, Sonic Hedgehog (SHH) in CAF-derived EVs can promote the proliferation and migration of esophageal squamous cell carcinoma (ESCC)through Hedgehog signaling^[32]^. Additionally, Valbona Luga *et al*. found that tumor cells endocytose CD81-containing EVs from CAFs, which could activate the Wnt/β-catenin pathway to promote metastasis^[33]^.

Accumulating evidence has recently shown that exosome-derived ncRNAs are vital for tumor progression^[34]^. ncRNAs mainly include miRNAs, lncRNAs, and circRNAs. They mainly bind to the 3’ untranslated region of target mRNA, inhibiting the translation and expression of target genes, thereby affecting the incidence and development of tumors^[35,36]^.

Metabolites derived from EVs affect the behavior of malignant tumors, and they can be divided into three major categories (1) amino acids: glutamine, threonine, serine, and valine; (2) lipids: palmitate and stearate; (3) tricarboxylic acid cycle intermediates: citrate, pyruvate, α-ketoglutarate, fumarate, and malate^[37]^. Tumor metabolic reprogramming is one of the important features of cancer^[38]^, with the upregulation of glycolysis, glutaminolysis, lipid metabolism, mitochondrial biogenesis, pentose phosphate pathway, and other biosynthetic and bioenergetic pathways^[39]^, which leads to the rapid proliferation, survival, invasion, metastasis, and resistance to treatment of tumor cells^[40]^.

### EVs from NFs and CAFs

In physiological conditions, fibroblasts are the quintessential supporting cell type and usually quiescent as shown by their low levels of cell proliferation and metabolic activity. They are present in all tissues, where they adapt to unique microenvironmental cues and cater to the needs of the specialized surrounding cells to help control tissue homeostasis and organ function. NFs in normal tissues produce ECM, which is vital to maintaining structural support and positional information for neighboring cells and offering a medium for cytokines, growth factors, and metabolites^[5,9,10]^. During tissue injury or inflammatory response, fibroblasts can be activated, with enhanced cell proliferation and metabolic activity, including protein synthesis. Activated fibroblasts are one of the key effector cell types in the wound healing process, which presumably promote wound healing via secreted factors including EVs^[41,42]^. Oh *et al*. successfully isolated and purified L929-EV from fibroblasts. It can accelerate wound healing in the mouse model of skin trauma by promoting fibroblast proliferation and migration, along with endothelial cell migration and lumen formation^[43]^. Xia *et al*.’s research found that EVs isolated from NFs at the wound edge of young mice were rich in miRNA-125b, which could accelerate myofibroblast differentiation and wound healing by inhibiting sirtuin 7^[44]^.

In certain conditions, the NFs can establish self-activating and feedback loops and transform into CAFs, which is crucial for the survival, proliferation, and invasion of cancer cells. For example, EVs secreted by CAFs differ in characteristics and biological functions from those secreted by NFs^[10]^. Specifically, EVs from CAFs typically carry large amounts of inhibitory cytokines (e.g., TGF-β) and death receptor ligands (e.g., PD-L1)^[45]^. Hu *et al*.’s study found that, compared with the EVs secreted by NFs, those secreted by CAFs increased expression of miR-92a-3p activates the Wnt/β-catenin pathway and inhibits mitochondrial apoptosis by directly inhibiting FBXW7 and MOAP1, contributing to cell stemness, EMT, metastasis, and 5-FU/L-OHP resistance in colorectal cancer (CRC)^[46]^. Additionally, Wang *et al*. found that in head and neck squamous cell carcinoma (HNSCC), CAF-derived EVs contain lower levels of miRNA-3188 compared to those derived from NFs^[47]^. In the TME, CAF-derived EVs also regulate tumor growth, metastasis, and angiogenesis, and mediate therapy resistance of tumor cells.

## REGULATORY MECHANISMS OF CAF-DERIVED EVS

### CAF-derived EVs regulate tumor proliferation

**Figure 2:**
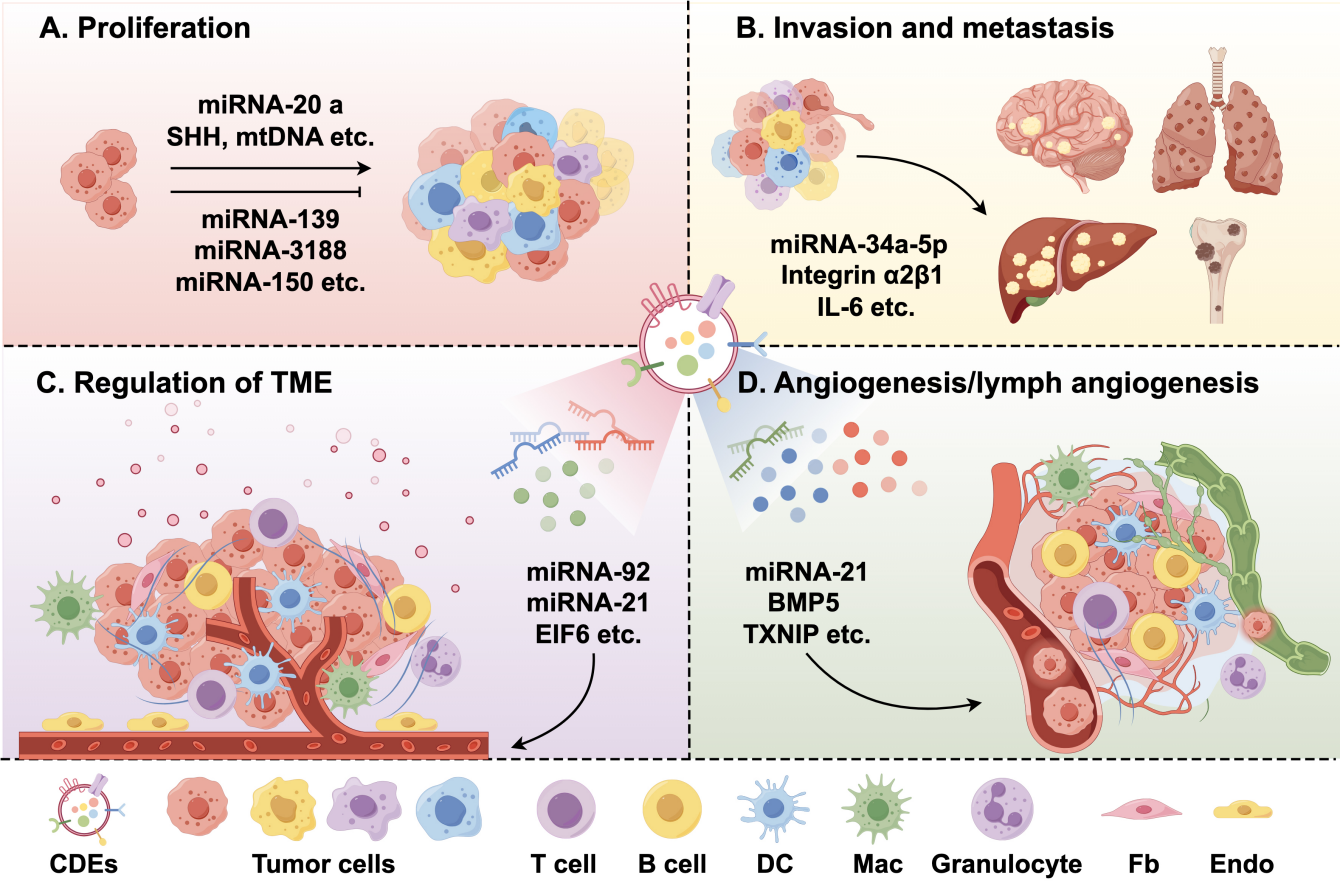
Contents and functions of CAF-derived EVs. (A) CAF-derived EVs regulate tumor proliferation; (B) CAF-derived EVs regulate tumor invasion and metastasis; (C) CAF-derived EVs regulate the tumor immune microenvironment; (D) CAF-derived EVs regulate tumor angiogenesis/lymphangiogenesis. CAF: Cancer-associated fibroblasts; EVs: extracellular vesicles; DC: dendritic cell; Mac: macrophage; Fb: fibroblast; Endo: endotheliocyte; TME: tumor microenvironment; IL-6: interleukin-6; CDEs: CAF-derived EVs.

The capacity for unlimited cell division is a cardinal feature distinguishing malignant tumor cell populations from their benign counterparts^[38]^. Accumulating evidence suggests that ncRNAs, particularly miRNAs, regulate tumor cell proliferation through modulation of signaling pathways^[48]^ [Figure 2A]. For instance, miRNA-20a has been found to inhibit the PTEN/PI3K-AKT pathway in non-small cell lung cancer (NSCLC), which promotes the proliferation of tumor cells^[49]^. Transfer of miRNA-500a-5p from CAFs to cancer cells in breast cancer (BC) stimulates proliferation and metastasis through binding to ubiquitin-specific peptidase 28 (USP28)^[50]^. Furthermore, it has been reported that under nutrient deprivation or nutrient stress conditions, CAF-derived EVs might inhibit mitochondrial oxidative phosphorylation and enhance glutamine-dependent reductive carboxylation and glycolysis, which promotes tumor growth^[40]^.

In addition to promoting the proliferation, certain substances in EVs can also inhibit the proliferation. A study by Xu *et al*. discovered that miRNA-139 in gastric cancer (GC) can inhibit development and metastasis by reducing the levels of matrix metalloproteinase 11 (MMP11) in the TME^[51]^. Wang *et al*. found that CAF-derived EVs in HNSCC contain lower levels of miRNA-3188 compared to those derived from NFs^[47]^. Similarly, in hepatocellular carcinoma (HCC), miRNA-150-3p^[52]^ and miRNA-320a^[53]^ in EVs have been found to be significantly reduced. The reduction in or absence of these miRNAs has been shown to promote tumor proliferation, invasion, and metastasis. Therefore, the potential therapeutic value of CAF-derived EVs in inhibiting tumor growth warrants further investigation.

### CAF-derived EVs regulate tumor invasion and metastasis

Metastasis is a significant cause of poor prognosis and mortality in cancer patients. It involves the dissemination of tumor cells to distant tissues and subsequent adaptation and survival in new microenvironments. Generally, the process of metastasis consists of four stages: local invasion and intravasation, survival in circulation, extravasation, and ultimately colonization at a new location^[54,55]^. Among the most critical strategies for metastasis is the promotion of tumor dissemination through EV-ncRNAs [Figure 2B].

In OSCC, the miR-34a-5p/AXL axis promotes β-catenin’s nuclear translocation and induces the EMT, which in turn leads to SNAIL’s transcriptional upregulation and the activation of MMP-2 and MMP-9^[56]^. The upregulation of MMP expression accelerates cancer invasion and metastasis, as well as poor prognoses^[57]^. Wu *et al*. found that the knockdown of the focal adhesion kinase (FAK) gene in CAFs promoted the migration of tumor cells. Analysis of miRNAs within CAF-derived EVs revealed multiple changes in EV-ncRNAs in FAK-deficient CAFs, such as the miRNA-16 and miRNA-148a. It is the upregulation of miRNA-16 and miRNA-148a that promotes the invasion and metastasis^[58]^. However, the underlying mechanisms remain unclear.

Additionally, research indicates that in the early stages of cancer, CAF-derived EVs may facilitate the establishment of a pre-metastatic microenvironment (PMN), thereby enhancing the likelihood of metastatic tumor cells successfully surviving and colonizing in foreign microenvironments. In salivary adenoid cystic carcinoma (SACC), CAF-derived EVs with upregulation of plasma IL-6 and integrin β1 possess robust stromal remodeling capabilities, inducing the formation of PMN in the lungs of mice and increasing pulmonary metastasis of SACC. Mechanistically, IL-6 activates the JAK2/STAT3 signaling pathway, which promotes EMT in SACC, thereby forming PMN to facilitate tumor metastasis^[59]^. In contrast, integrin β1 promotes the uptake of CAF-derived EVs by lung fibroblasts, further facilitating the formation of pulmonary PMN^[14]^. These findings enhance our understanding of the mechanisms underlying tumor invasion and metastasis, offering novel insights for future research in the domain of CAF-derived EVs.

### CAF-derived EVs regulate the TME

The inhibitory TME plays a pivotal role in determining the fate of tumors. During the initial stages of tumor development, the immune system can act as an antitumor defense. However, in later stages, tumor cells develop various mechanisms to evade immune surveillance. Despite recent advances in immunotherapy, the ability of tumors to evade immune surveillance has consistently been recognized as a barrier to the success of tumor immunotherapy [Figure 2C]^[60]^.

Researchers have shown that CAF-derived EVs can also contribute to the creation of an immunosuppressive microenvironment. Specifically, they can exhaust T cells in tumors and hinder their ability to perform essential immune functions. Feng *et al*. demonstrated that CAFs-EVs facilitate immune evasion in bladder cancer (BLCA) by upregulating the PD-L1/PD-1 pathway. Specifically, CAFs-EVs not only decrease apoptosis and enhance invasion in BLCA cells but also impair CD8+ T cell function by delivering PD-L1, thereby reducing CD8+ T cell proliferation and infiltration. Additionally, CAFs-EVs lower the secretion of key cytokines such as IFN-γ, IL-2, and TNF-α from CD8+ T cells^[61]^. Furthermore, through STAT3 activation, miRNA-21 in CAF-derived EVs promotes the production of MDSCs. MDSCs are a type of immunosuppressive cells capable of inhibiting the proliferation of CD8+ T cells and contributing to the establishment of an immunosuppressive microenvironment^[62,63]^.

On the other hand, Wang *et al*.’s study revealed that EVs in oral squamous cell carcinoma (OSCC) not only reduce CD3+ and CD8+ immune cells in tumor tissue, but also shape an immunosuppressive microenvironment by influencing the expression of immune genes. CAF-derived EVs contain numerous substances related to immune regulation, including has-miRNA-139-5p, EIF6, and PECAM1 miRNA, which regulate the expression of target cell proteins, including PIGR, CD81, UACA, and PTTG1IP, in cancer-related pathways, playing a significant role in regulating immune responses and promoting OSCC growth^[64]^. Currently, there is limited research on the impact of EVs on immune cells in the TME. Nevertheless, these studies provide a fresh perspective on the role of CAF-derived EVs in tumors and comprehensively reveal the immune microenvironment regulated by CAFs. Investigating the mechanisms of action of CAF-derived EVs in the tumor immune microenvironment represents a promising avenue for predicting prognosis and developing cancer treatment strategies.

### CAF-derived EVs regulate tumor angiogenesis/lymphangiogenesis

Tumors proliferate rapidly and require new blood/lymphatic vessel networks to obtain nutrients for growth, but this can also increase the risk of metastasis^[65]^. It has been demonstrated that CAF-derived EVs are crucial in regulating angiogenesis and lymphangiogenesis [Figure 2D].

In CRC, CAFs can downregulate the levels of BMP5 and TXNIP by upregulating the levels of miRNA -522-3p in small EVs^[66]^. This promotes proliferation, migration, and invasion while inhibiting CRC cell apoptosis *in vitro*. Similarly, Wu *et al*. demonstrated that downregulation of miRNA-29b-1-5p in CAF-derived EVs via the VSIG1/ZO-1 axis inhibits angiogenesis, GC cell migration and invasion, as well as the progression^[67]^. In the study conducted by Sun *et al*., it is noteworthy that miRNA-21 is highly abundant in CAF-derived EVs. Moreover, miRNA-21 has been shown to significantly upregulate the expression of fibroblast activation protein and α-smooth muscle actin in NFs, thereby inducing their transformation into CAFs. The CAF-derived EVs play a crucial role in promoting angiogenesis in multiple myeloma (MM) by delivering miRNA-21 to MM endothelial cells^[68]^.

In addition to regulating angiogenesis, CAF-derived EVs are also closely linked to the development of lymphatic vessels. For example, ESCC primarily affects the stomach and intestines and has a high potential for early lymphatic metastasis. Compared to EVs from NFs, CAF-derived EVs show significantly lower levels of miRNA-100-5p, which promotes the proliferation, migration, invasion, and lymphangiogenesis of tumor-associated lymphatic endothelial cells (TLECs). Mechanistically, miRNA-100-5p’s inhibitory effect on lymphangiogenesis may be mediated through the IGF1R/PI3K/AKT signaling axis^[69]^. Based on these findings, it has been observed that CAFs have the capability to secrete specific exosomes that regulate angiogenesis and lymphangiogenesis, thus opening up new avenues for research in this field.

Conclusively, CAF-derived EVs exert a pronounced regulatory influence on tumor initiation, progression, invasion, metastasis, immune evasion, and angiogenesis. The molecules, their mechanisms of action within these processes, and their biological functions are summarized in Table 1.

**Table 1:** The role and regulatory mechanism of CAF-derived EVs

**Cancer type**	**Molecule**	**Expression**	**Mechanism**	**Function**	**Ref.**
BC	miRNA-6, miRNA-148a	Upregulated	Through the FAK signaling pathway	Promotes invasion and metastasis	[58]
BC	miRNA-500 a-5p	Upregulated	Binds to USP28	Promotes proliferation and metastasis	[50]
BC	mtDNA	Upregulated	Restores oxidative phosphorylation of cancer cells, and activates cancer stem-like cells	Endocrine therapy resistance	[40]
CRC	miRNA-522-3p, BMP5, TXNIP	Upregulated	Inhibits the apoptosis, promotes angiogenesis	Promotes proliferation, migration, and invasion	[66]
ESCC	miRNA-100-5p	Downregulated	Inhibits lymphangiogenesis through IGF1R/PI3K/AKT axis	Inhibit lymphatic metastasis	[69]
ESCC	miR-21	Upregulated	Activates STAT3 signal transduction, induces MDSCs, inhibits CD8+ T cell proliferation	Establishes the immunosuppressive TME	[62,63]
ESCC	SHH	Upregulated	Activates the SHH signaling pathway	Promotes proliferation	[32]
GC	miRNA-139	Downregulated	Decreases MMP11 in the TME	Inhibits the metastasis	[51]
GC	miRNA-29b-1-5p	Upregulated	Promotes angiogenesis through the VSIG1/ZO-1 axis	Promotes proliferation, migration, and invasion	[67]
HCC	miRNA-150-3p, miRNA-320 a	Downregulated	Suppresses the MAPK pathway, inhibits EMT, decreases CDK2 and MMP2	Inhibits proliferation, migration, and invasion	[52,53]
HNSCC	miRNA-3188	Downregulated	Targets BCL2	Inhibits proliferation and promotes apoptosis	[47]
LC	Integrin α2β1	Upregulated	Promotes lung fibroblast activation and the formation of lung PMN	Creates a pre-metastatic niche, promotes the metastasis	[14]
LC	miRNA-20 a	Upregulated	Inhibits the PTEN/PI3K/AKT pathway	Promotes the proliferation	[49]
MM	miRNA-21	Upregulated	Transforms NFs into CAFs, promotes tumor neovascularization	Promotes invasion	[68]
OSCC	has-miRNA-139-5p, EIF6, PECAM1 mRNA	Upregulated	Affects the expression of immune genes, reduces CD3 and CD8+ immune cells	Establishes the immunosuppressive TME and promotes the proliferation	[64]
OSCC	miRNA-34a-5p	Upregulated	Activates the AKT/GSK-3β/β-catenin signaling pathway, induces EMT	Promotes progression and metastasis	[56,57]
SACC	Plasma integrin b1, IL-6	Upregulated	Activates the JAK2/STAT3 signaling pathway, induces EMT	Promotes invasion	[59]

BC: Breast cancer; BLCA: bladder cancer; CRC: colorectal cancer; ESCC: esophageal squamous cell carcinoma; GC: gastric cancer; HCC: hepatocellular carcinoma; HNSCC: head and neck squamous cell carcinoma; LC: lung cancer; MM: multiple myeloma; OSCC: oral squamous cell carcinoma; SACC: salivary adenoid cystic carcinoma; CDK2: cyclin-dependent kinase 2; BCL2: B-cell lymphoma 2; FAK: focal adhesion kinase; MDSCs: myeloid-derived suppressor cells; CAFs: cancer-associated fibroblasts; TME: tumor microenvironment; NFs: normal fibroblasts; PMN: pre-metastatic microenvironment; SHH: Sonic Hedgehog; USP28: ubiquitin-specific peptidase 28; MMP2: matrix metalloproteinase 2; MAPK: mitogen-activated protein kinase; EMT: epithelial-mesenchymal transition; PD-L1:programmed cell death ligand 1.

## POTENTIAL CLINICAL APPLICATIONS OF CAF-DERIVED EVS

### Innovative tumor biomarkers

EVs hold significant potential for liquid biopsy, as the cargo of EVs derived from tumor cells not only reflects the physiological and pathological characteristics of the tumor, but also remains stable in the body fluid circulation even within the harsh TME. Consequently, proteins and nucleic acids encapsulated in exosomes can serve as early diagnostic markers for cancer. For instance, miRNA-10b enclosed within exosomes is a well-established indicator of pancreatic adenocarcinoma (PAAD) progression and is widely used for early diagnosis. Furthermore, Glypican-1 (GPC1) is specifically enriched in exosomes, and circulating exosomes with high GPC1 expression can be highly specific and sensitive when detected in the serum of PAAD patients, distinguishing between early-stage and late-stage patients with PAAD as well as healthy individuals with pancreatic diseases^[70]^. For example, Tian *et al*. analyzed the cancer-related proteome of plasma EVs from BC patients and found that the EV features were highly accurate in differentiating between metastatic and non-metastatic BC. It can precisely monitor the response to treatment and serve as an independent prognostic factor in metastatic breast cancer (MBC) patients^[71]^. The development of biomarkers based on EVs is intrinsically linked to the advancement of EV detection technology. When contrasted with traditional EV analysis methods, such as polymerase chain reaction (PCR), western blot (WB), EV detection methods based on fluorescence, surface-enhanced Raman spectroscopy (SERS), surface plasmon resonance (SPR), electrochemistry, and aptamers enhance the sensitivity, specificity, and practicality of EVs in clinical settings^[72,73]^. In conclusion, as technology progresses and research intensifies, EVs will exhibit an even more significant potential in cancer.

### Drug delivery system in cancer therapy

In the field of cancer treatment, EVs are capable of enhancing the targeting capacity of cells or organs through genetic engineering and chemical modification and transferring various cargos to the target cells. The EV-mediated drug delivery technology is categorized into two types: exogenous and endogenous. Exogenous drug delivery technology refers to the isolation and purification of exosomes from cell culture supernatants or other biological fluids and the loading of the desired drugs onto the surface or within exosomes by physical or chemical means^[74]^. For instance, Santos *et al*. loaded exogenous miRNA-195-5p onto EVs. These modified EVs were absorbed by tumor cells and could reduce cell proliferation, increase cell death, and enhance targeted therapy responses in melanoma patients^[74,75]^. Exogenous loading is applicable when the ideal carrier is a small molecule drug or other molecules that cells cannot produce. The modification methods for EVs include passive incubation with cargo or active loading through electroporation, sonication, or freeze-thaw cycles. Meanwhile, endogenous drug delivery technology encompasses modifying donor cells via biotechnology to yield exosomes with specific products. This approach enables more precise control of the carrier content and has been employed to load therapeutic proteins, RNA, and CRISPR/Cas9 complexes into EVs^[74]^. For example, Katakowski *et al*. utilized a miRNA-146b plasmid to transfect MSCs, resulting in exosomes carrying it, which were then administered into the glioblastoma sites in mice. Their findings indicated that exosome-based therapy utilizing miRNA transport could effectively impede tumor cell proliferation^[76]^. Furthermore, numerous methods have been devised for the loading of siRNA into EVs, such as calcium chloride-mediated transfection, electroporation, sonication, and the employment of hydrophobically modified siRNA. For instance, in the study by Zhang *et al*., L9-29 mouse fibroblasts were transfected with TGF-β1 siRNA through a transfection reagent, and exosomes with TGF-β1 siRNA were isolated from the cell culture supernatant. The findings showed that compared with the use of free TGF-β1 siRNA, EVs carrying TGF-β1 siRNA effectively reduced the level of TGF-β1 in the targeted tumor cells, significantly lowering the viability and migration ability of mouse sarcoma cells^[77]^. The engineered CAF-derived EVs investigated by Xue *et al*. exhibit robust intercellular communication, transport, penetration, and targeting properties, making them a highly efficient delivery system capable of effectively overcoming the complexity and heterogeneity of the TME^[78]^. Similarly, there is significant potential in blocking tumor progression by regulating the secretion or level of CAF-derived EVs. For instance, the study by Sun *et al*. demonstrated that the application of anti-miRNA-21 or siRNA technology to lower the miRNA-21 level in CAF-derived EVs could diminish the proliferative, invasive, and metastatic capabilities of tumor cells *in vitro*^[68]^. Although the research targeting CAF-derived EVs for tumor treatment is still primarily at the animal experiment stage, it remains a potential target for cancer therapy.

### Advantages and disadvantages of EVs in clinical application

EVs have shown advantages and potential in clinical applications. Firstly, EVs have a natural targeting ability, as different cell-derived EVs interact with receptor carriers through surface adhesion proteins and selectively target receptor cells through their membrane components. For example, integrin α6β4 and α6β1 can target EVs to the lungs, while integrin α6β5 can promote EV targeting to the liver^[79,80]^. Secondly, EVs can be derived from the patient’s own cells, such as MSCs, thereby reducing their risk of immune rejection. Moreover, EVs are incapable of self-replication, which might render them safer than stem cell transplantation in regenerative medicine^[81,82]^. EVs possess distinctive advantages as biomarkers for therapeutic responses or drug delivery systems. EVs encompass various biological macromolecules and play a crucial role in information transmission among different cell types. They exhibit advantages such as stability, rich content, minimally invasive sampling, higher concentrations, and enhanced stability in liquid samples, as well as existence in various types of liquid samples. This renders EVs a promising new circulating biomarker with extensive application prospects^[72,83]^. Additionally, EVs possess the following advantages as drug delivery vehicles. Firstly, compared to cell therapy, exosomes are safer and easier to store, and they can be separated from the patient’s body fluids and modified before reintroduction, significantly reducing the likelihood of immune reactions occurring in clinical settings^[84,85]^. Secondly, exosomes possess the ability to enter the cell cytosol and protect drugs (such as nucleic acids) from metabolic processes during transportation, thereby extending the drug’s circulation time and improving its stability^[84]^. Thirdly, exosomes are nanometer-sized vesicles that carry cell surface molecules, giving them strong penetration and inherent targeting capabilities across various biological barriers^[86]^.

However, EVs have disadvantages in clinical applications. First, the half-life of EVs in body circulation is short, which might affect their sustained efficacy. The impact of EVs on tissues depends on their clearance rate, and macrophages have a certain clearance effect on EVs^[15,83,87]^. Regarding production efficiency, for regenerative therapy, EV production requires cultivating a large number of MSCs; however, the output of EVs secreted by cells is too low to meet therapeutic needs^[81,82]^. In terms of separation, purification, and storage, EVs have certain limitations. The currently mature methods for EV separation and purification include ultracentrifugation, immunoprecipitation or affinity purification, ultrafiltration, and size exclusion; however, the purity of purified EVs is often low, affecting subsequent experiments. The optimal storage conditions for maintaining Evs’ integrity (such as temperature or pH value) are rather demanding^[15,72,83]^. Nonetheless, EVs continue to demonstrate significant potential for precision cancer treatment in the future.

## CONCLUSION

CAF-derived EVs have a multifaceted and pivotal impact on the TME. This comprehensive review outlines the diverse functions of CAF-derived EVs in tumorigenesis, progression, metastasis, and immune evasion. It also investigates their potential applications in cancer therapy.

Firstly, CAF-derived EVs influence tumor cell proliferation, invasion, and metastasis by transporting various biologically active molecules such as proteins, nucleic acids, and metabolic products. Notably, ncRNAs such as miRNAs and lncRNAs modulate the expression of target genes, enhancing tumor cells’ stem-like properties and EMT while improving chemoresistance and metastatic potential. Additionally, metabolic products in CAF-derived EVs contribute to reprogramming tumor metabolism by providing energy and precursor molecules for tumor growth and invasion. Secondly, CAF-derived EVs play a critical role in regulating the TIME. They can impact T cell function, facilitate MDSCs generation, and regulate the expression of immune-related genes to establish an immunosuppressive microenvironment that aids tumors in evading immune surveillance. Furthermore, the role of CAF-derived EVs in tumor angiogenesis and lymphangiogenesis warrants significant attention. By delivering specific miRNAs, CAF-derived EVs can modulate the expression of factors associated with angiogenesis, thereby impacting the vascular supply and metastatic potential of tumors.

In terms of clinical application, circulating CAF-derived EVs have shown promise as novel biomarkers, owing to their stability in bodily fluids and ability to reflect tumor characteristics through specific proteins and nucleic acids. Additionally, CAF-derived EVs can function as delivery vehicles to effectively transport therapeutic molecules to tumor cells. However, improving the targeting and purity of CAF-derived EVs remains a significant challenge in realizing their clinical utility. Lastly, it is possible to inhibit tumor proliferation and metastasis by modulating the secretion of CAF-derived EVs or the levels of their constituents, thereby providing a new avenue for therapeutic intervention.

In summary, the role of CAF-derived EVs in tumor biology is multifaceted, with broad prospects for their application in tumor diagnosis and treatment. Future research needs to further elucidate the specific mechanisms of action of CAF-derived EVs, optimize their use as biomarkers and drug carriers, and explore new targeted therapies for more effective cancer treatments.

## DECLARATIONS

### Copyright

© The Author(s) 2025.
